# Design and Synthesis of Chalcone Derivatives as Inhibitors of the Ferredoxin — Ferredoxin-NADP^+^ Reductase Interaction of *Plasmodium falciparum*: Pursuing New Antimalarial Agents

**DOI:** 10.3390/molecules191221473

**Published:** 2014-12-19

**Authors:** Hery Suwito, Jumina, Mustofa, Pratiwi Pudjiastuti, Much Zaenal Fanani, Yoko Kimata-Ariga, Ritsuko Katahira, Toru Kawakami, Toshimichi Fujiwara, Toshiharu Hase, Hasnah Mohd Sirat, Ni Nyoman Tri Puspaningsih

**Affiliations:** 1 Department of Chemistry, Faculty of Science and Mathematics, University of Gajah Mada, Jogjakarta 55281, Indonesia; 2 Department of Chemistry, Faculty of Science and Technology, Airlangga University, Surabaya 60115, Indonesia; 3 Department of Pharmacology and Therapy, Faculty of Medicine, University of Gajah Mada, Jogjakarta 55281, Indonesia; 4 Institute of Tropical Disease, Airlangga University, Surabaya 60115, Indonesia; 5 Institute of Protein Research, Osaka University, 3-2 Yamadoaka, Suita-Shi, Osaka 656-0871 Japan; 6 Department of Chemistry, Faculty of Science, University Technology Malaya, Johor Bahru 81310, Malaysia

**Keywords:** methoxyamino chalcones, antimalarial, *Pf*Fd-*Pf*FNR inhibitor

## Abstract

Some chalcones have been designed and synthesized using Claisen-Schmidt reactions as inhibitors of the ferredoxin and ferredoxin-NADP^+^ reductase interaction to pursue a new selective antimalaria agent. The synthesized compounds exhibited inhibition interactions between *Pf*Fd-*Pf*FNR in the range of 10.94%–50%. The three strongest inhibition activities were shown by (*E*)-1-(4-aminophenyl)-3-(4-methoxyphenyl)prop-2-en-1-one (50%), (*E*)-1-(4-aminophenyl)-3-(2,4-dimethoxyphenyl)prop-2-en-1-one (38.16%), and (*E*)-1-(4-aminophenyl)-3-(2,3-dimethoxyphenyl)prop-2-en-1-one (31.58%). From the docking experiments we established that the amino group of the methoxyamino chlacone derivatives plays an important role in the inhibition activity by electrostatic interaction through salt bridges and that it forms more stable and better affinity complexes with FNR than with Fd.

## 1. Introduction

Malaria is considered a global health problem. In year 2012, there were an estimated 207 million malaria cases and the disease was responsible for 627,000 deaths [1]. Although numerous antimalarial drugs have been used, their applications are limited due to toxicity and resistance [2]. High rates of resistance of *Plasmodium falciparum* to sulfadoxine-pyrimethamin (SP) and chloroquine have been identified in several endemic areas in Indonesia [3]. Mutations in the *pfdhfr* and *pfdhps* genes of *Plasmodium falciparum* isolated from Banjar district, South Kalimantan Province, Indonesia associated with the SP resistance has recently been reported [4]. Resistance to Artemisinin-based Combination Therapies (ACTs) has also already been reported in four countries of the greater Mekong subregion: Cambodia, Myanmar, Thailand, and Vietnam [1]. Based on the mentioned situation, the need for new antimalarial agents has motivated the research to find synthetic molecules which are able to answer the problem.

*Plasmodium falciparum* possesses a non-photosynthetic plastid organelle called apicoplast which is very important for the parasite’s survival. This respiration organelle is *Plasmodium falciparum*-specific and it is not present in humans [5]. During the respiration process, electron transfer to ferredoxin (*Pf*Fd) catalyzed by ferredoxin NADP^+^ reductase (*Pf*FNR) occurs in the apicoplast [6]. The interaction of *Pf*FNR-*Pf*Fd proceeds through an intermolecular electrostatic interaction between acidic amino acid residues of *Pf*Fd with basic amino acid residues of *Pf*FNR. There are two acidic regions of *Pf*Fd that dominantly contribute to the electrostatic interaction with *Pf*FNR, those are Asp26, Glu29, Glu34, and the others include Asp65 and Glu66 [7]. Therefore targeting inhibition of the interaction of these proteins could open new possibilities to find new and selective antimalarial compounds.

Chalcones are natural products that can also be obtained synthetically using a relatively simple synthesis procedure. The general method applied to synthesize chalcones is the Claisen-Schmidt reaction, while a modern alternative to synthesize chalcones uses the palladium-catalyst cross coupling reactions of styryltrifluoroborates with benzoyl chlorides [8]. Chalcone derivatives are well known for their broad spectrum of pharmacological activities, inclusing radical scavenger [9], antihepatotoxic [10], anticancer [11], and antimalarial properties [12]. Alkoxylated chalcone derivatives exhibited higher antimalarial activity compared to hydroxylated chalcones [13]. 

The synthesis and bioactivity of the prepared compounds were already reported. The interaction of compounds **1**‒**3** with bovine serum albumin (BSA)—a protein mainly responsible for the transportation of a number compounds in a living system—has been studied, and it was found that compound **1** was the most reactive [14]. Furthermore, compound **3** was used as intermediate in the synthesis of phthalimide derivatives as analgesic and anti-inflammatory agents [15]. Continuous-flow hydration-condensation reactions between phenylacetylene and benzaldehyde derivatives using Amberlyst 15 as heterogeneous catalyst was applied for the synthesis of compounds **8**, **10**, and **12** in excellent yield [16]. Compound **10** exhibited good anticancer activity toward the A549, PC3, MCF-77, HT-29, and WRL68 cancer cell lines [17], while compound **12** showed potential anticancer, anti-inflammatory, and antioxidant activity [18]. Based on the background, herein we report the synthesis of methoxychalcone derivatives designed to inhibit the *Pf*Fd-*Pf*FNR interaction and analyze the interaction through docking experiments. 

## 2. Results and Discussion

### 2.1. Design and Synthesis of Chalcone Derivatives

According to the electrostatic interaction between the acidic amino acid residues of *Pf*Fd with the basic amino acid residues of *Pf*FNR [7] and the important role of the methoxy group for antimalarial activity [13], we designed and then synthesized methoxyamino chalcone derivatives. The amino group is assumed to form an electrostatic interaction with the acidic amino acid residues of *Pf*Fd to hinder the *Pf*Fd-*Pf*FNR electrostatic interaction. To prove this assumption, we then synthesized methoxybromo chalcone derivatives, as bromo substituted on an aromatic group is known as an electron withdrawing group, while the amino group is known as an electron donating group. The role of the methoxy group was studied by synthesizing chalcone **17**. The synthesis of chalcone derivatives was accomplished using the Claisen-Schmidt reaction. This reaction can take place under various reaction conditions [12,13,19,20,21]. In our research, the chalcones were prepared by the reaction of equimolar amounts of acetophenone and benzaldehyde derivatives in ethanol using a 40% NaOH solution as catalyst. By applying these reaction conditions, good to excellent yields were obtained (Table 1).

**Table 1:** Structure of the prepared chalcones. 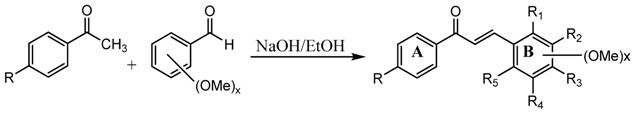

Compd	R	R_1_	R_2_	R_3_	R_4_	R_5_
**1**	NH_2_	OCH_3_	H	H	H	H
**2**	NH_2_	H	OCH_3_	H	H	H
**3**	NH_2_	H	H	OCH_3_	H	H
**4**	NH_2_	OCH_3_	OCH_3_	H	H	H
**5**	NH_2_	OCH_3_	H	OCH_3_	H	H
**6**	NH_2_	OCH_3_	H	H	OCH_3_	H
**7**	NH_2_	H	H	H	H	H
**8**	H	OCH_3_	H	H	H	H
**9**	H	H	OCH_3_	H	H	H
**10**	H	H	H	OCH_3_	H	H
**11**	H	OCH_3_	OCH_3_	H	H	H
**12**	H	OCH_3_	H	OCH_3_	H	H
**13**	H	OCH_3_	H	H	OCH_3_	H
**14**	Br	H	H	OCH_3_	H	H
**15**	Br	OCH_3_	H	OCH_3_	H	H
**16**	Br	OCH_3_	H	H	OCH_3_	H
**17**	H	H	H	H	H	H

The molecular structures of the synthesized compounds were confirmed by FT-IR, HRESI-MS or ESI-MS, and ^1^H- and ^13^C-NMR spectral data. The ^1^H-NMR coupling constant analyses indicated that all hydrogen atoms of the olefinic carbon–carbon bond were in a *trans* conformation. The existence of a carbonyl group conjugated with the olefinic carbon–carbon bond was evident from the infra-red spectra as the carbonyl peak was observed at a lower wavenumber than a normal carbonyl peak (around 1650–1660 cm^−1^) and from the ^13^C-NMR spectra. The existence of a bromine atom in compounds 14, 15 and 16 was detected by the appearance in their mass spectra of two molecular peaks in the [M+H]^+^ at *m/z* 317.3 and 319.2 (compound 14), 347.3 and 349.3 (compound 15), and at *m/z* 347.1 and 349.2 (compound 16) with an intensity of 1:1. The existence of methoxy groups was evidenced by the peaks at chemical shifts between 3.6 until 3.9 ppm in the ^1^H-NMR spectra [22].

### 2.2. Production, Characterization and Kinetic Analysis of PfFd and PfFNR

**Figure 1:**
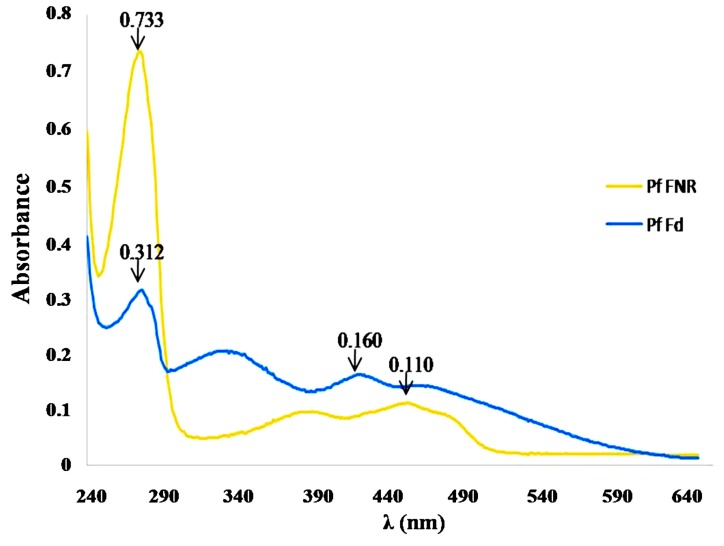
UV-Vis spectrum profile of the proteins *Pf*Fd and *Pf*FNR.

The protein *Pf*FNR was isolated from the clone *E coli* TG1 pTrc99A, whereas protein *Pf*Fd was produced from the clone *E coli* JM105 pTrc99A (both from the collection of the Laboratory of Regulation and Biological Reaction IPR Osaka University-Japan). Confirmation of protein purity was determined from their UV-Vis spectrum profiles at λ between 240–650 nm (Figure 1). 

The red colored *Pf*Fd showed peaks at 423, 334, and 276 nm, whereas the yellow colored *Pf*FNR showed peaks at 455, 389, and 276 nm, which indicated the specific peaks for the family protein containing 2Fe-2S and flavoprotein, respectively [6,7]. The absorbance ratios Abs_276/423_ and Abs_276/455_ were then used to determine the purity of each protein, which resulted in 1.95 for *Pf*Fd and 6.66 for *Pf*FNR. These values were close to the results from a previous report [7], 1.75 and 7.0. 

**Scheme 1:**
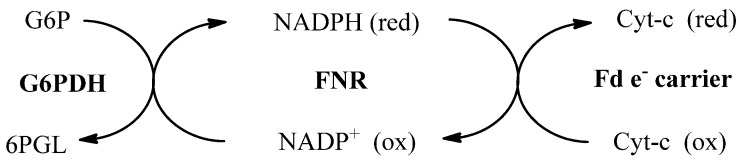
Chain reaction cycle applied in the inhibition assay.

The kinetic parameter determined in this study was the Michaelis constant (K_M_). The basis for the kinetic parameter determination procedure was in accordance with Scheme 1. The number of reduced Cyt-c is equivalent to the number of electrons transferred by *Pf*FNR from NADPH to *Pf*Fd, which was observed spectrometrically through the absorbance exchange by adding oxidized-Cyt-c as indicator at 550 nm. Glucose-6-phosphate dehydrogenase (G6PDH) and glucose-6-phosphate (G6P) were added as a NADPH regeneration system. The K_M_ value obtained from this research was 0.80 µM^−1^, which was the same value as the one reported before [6,23].

### 2.3. Inhibition of PfFd-PfFNR Interaction by Synthesized Compounds

The inhibition assay was performed under the same conditions used in the K_M_ determination employing the following conditions: the concentration of *Pf*Fd was 1 µM, the concentration of *Pf*FNR was 20 nM, and the concentration of tested compounds was 100 µM. A 5 mM solution of DMSO was used as control solution. The % inhibition was calculated according to the following equation, and the results are listed in Table 2:

(1) $$\%Inhibition=\;\frac{\left(\frac{dA}{dt}\right)control-\left(\frac{dA}{dt}\right)sample}{\left(\frac{dA}{dt}\right)control}\;\times \;100\%$$

**Table 2:** Inhibition (%) of electron transfer of the tested chalcones (100 µM) to *Pf*Fd.

Tested Compound	dA/dt	% Inhibition
**Control-1**	0.076	-
**1**	0.064	15.79
**2**	0.059	22.37
**3**	0.038	50.00
**4**	0.052	31.58
**5**	0.047	38.16
**Control-2**	0.064	-
**6**	0.073	−14.06
**7**	0.057	10.94
**10**	0.061	4.69
**12**	0.060	6.25
**Control-3**	0.068	-
**8**	0.075	−10.29
**9**	0.068	0.00
**11**	0.072	−5.88
**13**	0.064	5.88
**14**	0.063	7.35
**15**	0.050	26.47
**16**	0.060	11.76
**17**	0.053	22.06

The results showed that some of the tested compounds were able to inhibit the electron transfer by between 4.68%–50% (compounds **1**‒**5**, **7**, **10**, **12**‒**17**), while compound **9** showed no activity. Compounds **6**, **8**, and **11** exhibited **a** negative inhibition, which means that these compounds accelerated the electron transfer from *Pf*FNR to *Pf*Fd. In general, these results gave us the following information: (1) methoxyamino chalcone derivatives exhibited better inhibition of electron transfer than methoxybromo chalcones and methoxy chalcones with no amino group; (2) the amino group played an important role in the inhibition activity, whereas the existence of a methoxy group gave only minor contributions to the inhibition activity. The effect of *ortho*- or *meta*-substituted amino groups on ring A will be investigated in the future.

### 2.4. In Silico Analysis of the Protein—Synthesized Compounds Interaction

The molecular interaction between both proteins and synthesized compounds was studied by docking experiments. Because the molecular structure of *Pf*Fd-*Pf*FNR complex is not available in the Protein Data Bank (PDB), we took the 3D-structure of *Pf*Fd from PDB with access code 1IUE to study the interaction of the tested compounds with *Plasmodium falciparum* ferredoxin, whereas the interaction of *Pf*FNR with the synthesized compounds was studied by taking the 3D-structure of maize-FNR obtained from the structure of maize Fd-FNR complex (PDB access code 1GAQ) [24]. The 3D-structure of maize FNR was used for the docking experiment because of its high homology to the *Pf*FNR (82%) and it was obtained after the maize-Fd structure was deleted from the complex structure.

The downloaded structure of *Pf*Fd (1IUE) contained no ligand, so that it was impossible to define the binding position and run a docking validation, which was to be used subsequently in the docking experiment. Therefore, the grid box used for docking experiment was made as follow. The position of the grid box was derived from the position of the amino acid residues of *Pf*Fd involved dominantly in the interaction with *Pf*FNR; Asp26, Glu29, Glu34, Asp65, and Glu66 [7], while the dimension of grid box was made, so that it could cover all these amino acid residues. 

**Figure 2:**
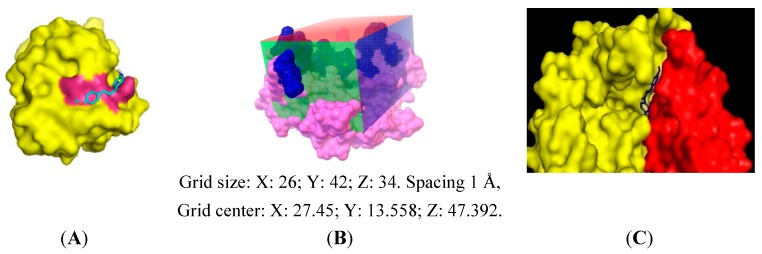
Docking result of compound **3** generated by AutoDock4 and viewed by PyMol. (**A**) Docking pose of compound **3** into *Pf*Fd. The magenta color is the surface of amino acid residues of *Pf*Fd involved in the interaction with *Pf*FNR, (**B**) Grid box used in docking experiment into maize leaf-FNR. Dark blue color is the surface of active amino acid residues in the interaction with maize leaf-Fd, (**C**) docking pose of compound **3** on maize-FNR and attached by maize-Fd. Yellow: maize leaf-FNR, red: maize leaf-Fd, and blue: compound **3**.

The same preparation was conducted for docking experiments to maize leaf-FNR. The position of the grid box was derived from the position of amino acid residues of maize leaf-FNR involved in the interaction with maize leaf-Fd; Lys88, Lys91, Lys33, Glu154, and Lys304 [24]. To verify the docking condition, a docking experiment of compound **3** to maize leaf-FNR was performed, and then the maize leaf-Fd were put to the docking result. The docking results are shown in Figure 2. The docking results of the synthesized compounds into *Pf*Fd and maize-FNR are tabulated in Table 3. The docking of compounds **14**‒**16** into both proteins was not performed. 

**Table 3:** The docking results of synthesized compounds into *Pf*Fd and maize-FNR.

Comp	Protein *Pf*Fd			Protein Maize Leaf-FNR		
	∆G Energy (Kcal/mol)	Amino Acid Residues Form H-Bonding	Amino Acid Residues Form Electrostatic Interaction	∆G Energy (Kcal/mol)	Amino Acid Residues Form H-Bonding	Amino Acid Residues Form Electrostatic Interaction
**1**	−2.45	Tyr37	---	−3.68	Asn30	Tyr32
**2**	−2.96	Arg30, Asn32	---	−3.78	Asn30, Leu94	Tyr32
**3**	−2.71	Arg30, Tyr37	Glu34	−4.00	Lys304	Lys304, Asp307
**4**	−2.45	Arg30,	Glu34	−3.50	Lys304, Arg305	Lys304, Asp307
**5**	−2.43	Asn32	---	−4.09	Lys304, Arg305	---
**6**	−2.49	Arg30, Tyr37	---	−3.55	Asn30, leu94, Thr148	---
**7**	−2.79	Tyr37	Glu34	−4.10	Lys304(2x)	Asp307
**8**	−2.47	Asn32, Tyr37	---	−3.99	Lys301, Lys304	---
**9**	−2.90	---	---	−4.33	Val311	---
**10**	−2.75	---	---	−4.09	Lys304, Arg305	---
**11**	−2.32	---	---	−3.84	Lys304	---
**12**	−2.48	---	---	−3.76	Lys304	---
**13**	−2.57	---	---	−4.09	Lys304	---
**14**	-	---	---	-	Not observed	---
**15**	-	---	---	-	Not observed	---
**16**	-	---	---	-	Not observed	---
**17**	−2.65	---	---	−4.03	Lys304	---

In general, the docking results provide us with the information that the affinity of synthesized compounds toward maize leaf-FNR was bigger than toward *Pf*Fd as shown by the lower maize leaf-FNR–synthesized compounds binding energy. Beside affinity, the inhibitory activity of the synthesized compounds can also be reflected from the interaction of the protein with the tested compounds (H-bonding, electrostatic interactions through salt bridges). 

### 2.5. Docking into PfFd

**Figure 3:**
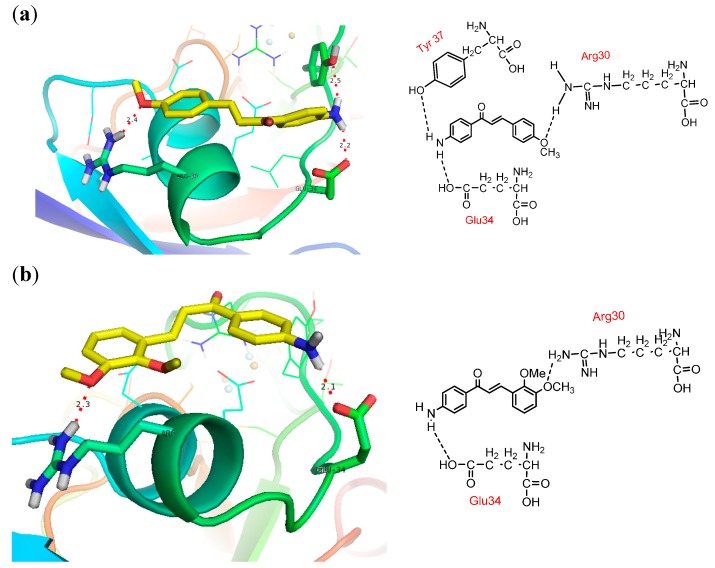

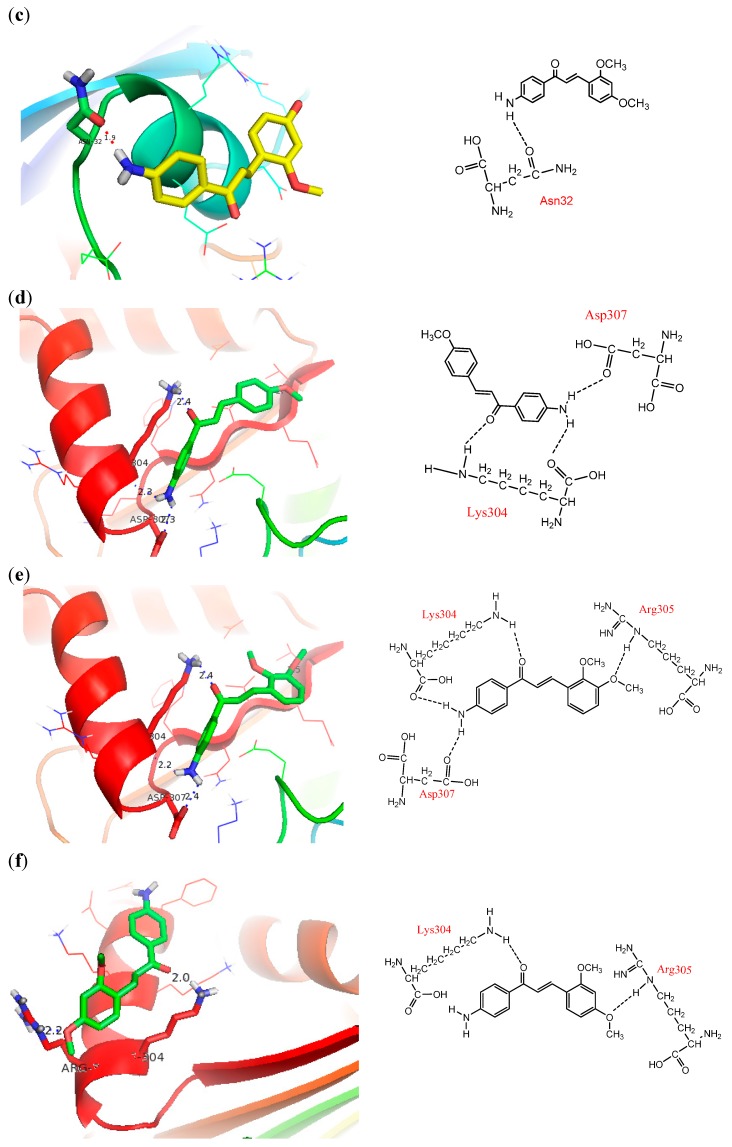
Docking pose and 2D presentation of the molecular interaction of the synthesized chalcones on *Pf*Fd and maize leaf-FNR. (**a**) *Pf*Fd-compound **3**, (**b**) *Pf*Fd-compound **4**, and (**c**) *Pf*Fd -compound **5**, (**d**) maize leaf-FNR-compound **3**, (**e**) maize leaf-FNR-compound **4**, (**f**) maize leaf-FNR-compound **5**.

Visualization of docking results employing *PyMol* program exhibited that all methoxyamino chalcone derivatives (compounds **1**–**7**) formed hydrogen bonds with amino acid residues of *Pf*Fd, while the others did not show hydrogen bond interactions. In this article we report only the visualization of the interactions of compounds **3**‒**5** that showed high inhibitory activity with *Pf*Fd and maize leaf-FNR, displayed in Figure 3. From the docking results the following observations were noted: (1) the acidic amino acid residue of *Pf*Fd involved in electrostatic acid-base interactions with the amino groups of the synthesized chalcones was Glu34. This result was in accordance with the results of a previous study which showed that the acidic region of *Pf*Fd conferred a larger contribution for electrostatic interactions [7]. With *Pf*FNR it was Asp26/Glu29/Glu34 [7]; (2) due to its dominant involvement of Tyr37 of *Pf*Fd in the formation of hydrogen bonds with the tested compounds, we assumed that this amino acid residue functions as modulator in the electron transfer process; (3) the amino groups of the tested compounds played an active role in the inhibition of electron transfer to *Pf*Fd. The amino group in the tested compounds was able to build electrostatic interactions with the acidic residue Glu34 of *Pf*Fd and consequently, the *Pf*Fd-*Pf*FNR interaction was inhibited; (4) the existence of a methoxy group played an insignificant role in the electron transfer process to *Pf*Fd (comparing the inhibition activity of compounds **1**–**7** to **8**–1**3** and **17**); (5) the presence of a bromine atom as an electron withdrawing group played a minor role in the transfer electron process; (6) however, for compounds **6**, **8**, and **11** which showed acceleration of transfer electron property, this still remains to be explored.

### 2.6. Docking into Maize Leaf-FNR

The docking results showed that the tested compounds formed more hydrogen bonding and electrostatic interactions with maize leaf-FNR than with *Pf*Fd. Particularly interesting is the electrostatic interaction between the amino groups of the tested compound with the carboxyl group of non-acidic amino acid residues of FNR which was observed in methoxyamino chalcone derivatives **1**–**7**. This can explain the inhibition mechanism of the tested compounds through inhibition of electron flow from FNR to Fd, which is in accordance with the electron flow during respiration process in the apicoplasts of *P falciparum*. Based on the docking results analysis, we assume that the basic amino groups of the synthesized compounds interact better with the active amino acid residues of FNR than with the active amino acid residues of Fd. This interaction leads to a good three dimensional complementarity between the FNR structure and the synthesized compounds.

## 3. Experimental Section 

### 3.1. General Information

The chemicals used in the research were pro analysis grade. Melting points were measured with a Fisher John melting point apparatus and are uncorrected. The purity of the synthesized compounds were checked by thin layer chromatography on silica gel GF_254_ plates; the eluent was a mixture of *n*-hexane/ethyl acetate in 3:2 ratio and the spots were identified by UV (254 nm). The mass spectra were recorded by HRESI-MS (Waters LCT Premier XE, Waters Corp., Milford, MA, USA) or on ESI-MS (LCQ-DECA XP Plus, Thermo-Finnigan, San Diego, CA, USA) spectrometers; NMR spectra were recorded in CDCl_3_ on a NMR Bruker 400 MHz instrument (400.13 MHz for ^1^H and 100.61 MHz for ^13^C, Bruker, Billerica, MA, USA). The IR spectra were recorded on a Spectrum One FT-IR spectrophotometer (Perkin Elmer, Waltham, MA, USA). The UV-Vis spectra were measured on a UV-2500 PC UV-Vis spectrophotometer (Shimadzu, Kyoto, Japan). The inhibition rates and enzyme activity were recorded on a microplate reader (SH-1000 Lab Microplate Reader, Corona Electric Co., Ltd., Ibaraki, Japan).

### 3.2. General Procedure for Chalcone Synthesis

A mixture of acetophenone derivative (6 mmol) and benzaldehyde derivative (6 mmol) was dissolved in ethanol (30 mL), then NaOH 40% solution (6 mL) was added dropwise, while the temperature was kept under 10 °C, the reaction mixture was stirred under this condition for 1 hour, then the stirring was continued at room temperature for 4 hours. Thereafter the reaction mixture was poured into ice-water; the precipitated solid was filtered off, and recrystallized from aqueous ethanol. For compound **17**, the synthesis was conducted according to procedure described by Durst and Gokel [19].

(*E)-1-(4-Aminophenyl)-3-(2-methoxyphenyl)prop-2-en-1-one* (**1**): yellow crystals (1.14 g; 75%), m.p 104–106 °C, Rf = 0.42 (*n*-hexane/ethyl acetate: 3/2); HRESI-MS [M+H]^+^ calculated for C_16_H_16_NO_2_ 254.1181, found 254.1180; IR (KBr, cm^−1^): 3445 and 3344 (-NH_2_); 1637 (C=O), 1596 (C=C_chain_), 1173 (C-O-C_aryl alkyl ether_); ^1^H-NMR (CDCl_3_) δ 8.11 (*d*, 1H, *J* = 15.6 Hz); 7.67 (*d*, 1H, *J* = 15.6 Hz); 7.01 (*t*, 1H); 7.38 (*s*, 1H); 6.97 (*s*, 1H,); 6.95 (*s*, 1H); 7.96 (*d*, 1H, *J* = 8.4 Hz); 6.72 (*d*, 1H, *J* = 8.8 Hz); 3.93 (*s*, 3H); 4.19 (*s*, br, 2H); ^13^C-NMR (CDCl_3_) δ 141.0; 121.3; 125.7; 159.2; 114.2; 128.9; 120.9; 135.2; 127.9; 132.0; 114.7; 154.2; 114.7; 132.0; 56.2; 189.70.

(*E)-1-(4-Aminophenyl)-3-(3-methoxyphenyl)prop-2-en-1-one* (**2**): yellow crystals (1.31 g; 86%), m.p 118–120 °C, Rf = 0.47 (*n*-hexane/ethyl acetate: 3/2); HRESI-MS [M+H]^+^ calculated for C_16_H_16_NO_2_ 254.1181, found 254.1181; IR (KBr, cm^−1^): 3439 and 3338 (-NH_2_), 1645 (C=O), 1610 (C=C_chain_), 1173 (C-O-C_aryl alkyl ether_); ^1^H-NMR (CDCl_3_) δ 7.77 (*d*, 1H, *J* = 15.6 Hz); 7.54 (*d*, 1H, *J* = 15.6 Hz); 7.17 (*t*, 1H, *J* = 2 Hz); 7.25 (*d*, 1H, *J* = 7.6 Hz); 7.35 (*t*, 1H, *J* = 8 Hz); 7.95 (*d*, 1 H, *J* = 8.8 Hz); 6.72 (*d*, 1H, *J* = 8.8 Hz); 7.95 (*d*, 1 H, *J* = 8.8 Hz); 3.88 (*s*, 3H), 4.21 (*s*, br, 2 H). ^13^C-NMR (CDCl_3_) δ 143.04; 122.44; 136.76; 115.83; 159.94; 113.36; 129.85; 120.91; 128.56; 131.10; 119.95; 151.14; 119.95; 55.35; 188.12.

(*E*)-*1-(4-Aminophenyl)-3-(4-methoxyphenyl)prop-2-en-1-one* (**3**): yellow crystals (1.138 g, 75%); m.p 140–142 °C; Rf = 0.42 (*n*-hexane/ethyl acetate: 3/2); HRESI-MS [M+H]^+^ calculated for C_16_H_16_NO_2_ 254.1181, found 254.1183; IR (KBr, cm^−1^): 3467 and 3328 (-NH_2_), 1631 (C=O), 1598 (C=C_chain_), 1162 (C-O-C_aryl alkyl ether_); ^1^H-NMR (CDCl_3_) δ 7.78 (*d*, 1H, *J* = 15.6 Hz); 7.46 (*d*, 1H, *J* = 15.6 Hz); 7.61 (*d*, 1H, *J* = 8.4 Hz); 6.72 (*d*, 1H, *J* = 8.4 Hz); 7.61 (*d*, 1H, *J* = 8.4 Hz); 7.95 (*d*, 1H, *J* = 8.8 Hz); 6.95 (*d*, 1H, *J* = 8.8 Hz); 7.95 (*d*, 1H, *J* = 8.8 Hz); 3.87 (*s*, 3H); 4.21 (*s*, br, 2H). ^13^C-NMR (106.61 MHz, CDCl_3_) δ 142.97; 119.81; 128.83; 129.96; 114.36; 161.33; 128.10; 130.96; 113.94; 150.99; 130.96; 55.59; 188.23.

(*E*)-*1-(4-Aminophenyl)-3-(2,3-dimethoxyphenyl)prop-2-en-1-one* (**4**): yellow crystals (1.358 g, 80%); m.p = 140–142 °C; Rf = 0.42 (*n*-hexane/ethyl acetate: 3/2); HRESI-MS [M+H]^+^ calculated for C_17_H_17_NO_3_ 283.1287, found 283.1286; IR (KBr, cm^−1^): 3466 dan 3427 (-NH_2_), 1631 (C=O), 1602 (C=C_chain_), 1169 (C-O-C_aryl alkyl ether_); ^1^H-NMR (CDCl_3_) δ 8.08 (*d*, 1H, *J* = 15.6 Hz); 7.63 (*d*, 1H, *J* = 15.6 Hz); 6.97 (*dd*, *J_1_* = 1.2 Hz; *J_2_* = 8.0 Hz); 7.11 (*t*, 1H, *J* = 8 Hz); 6.60 (*d*, 1H, *J_1_* = 1.2 Hz; *J_2_* = 8.0 Hz); 7.96 (*d*, 1H, *J* = 8.8 Hz); 6.72 (*d*, 1H, *J* = 8.8 Hz); 7.96 (*d*, 1H, *J* = 8.8 Hz); 3.91 (*s*, 3H); 3.89 (*s*, 3H); 4.16 (*s*, br, 2H). ^13^C-NMR (CDCl_3_) δ 131.6; 124.1; 137.9; 152.5; 153.2; 113.9; 123.8; 119.7; 129.6; 131.1; 113.9; 151.1; 61.3; 55.9; 188.5.

(*E)-1-(4-Aminophenyl)-3-(2,4-dimethoxyphenyl)prop-2-en-1-one* (**5**): yellow crystals (1.66 g, 98%); m.p 148–150 °C; Rf = 0.49 (CHCl_3_/ethyl acetate: 3/2); HRESI-MS [M+H]^+^ calculated for C_17_H_17_NO_3_ 283.1287, found 283.1288; IR (KBr, cm^−1^): 3425 and 3349 (-NH_2_), 1634 (C=O), 1597 (C=C_chain_), 1171 (C-O-C_aryl alkyl ether_); ^1^H-NMR (CDCl_3_) δ 8.05 (*d*, 1H, *J* = 15.6 Hz); 6.51 (*d*, 1H, *J* = 15.6 Hz); 7.58 (*d*, 1H, *J* = 1.2 Hz); 6.55 (*dd*, 1H, *J_1_* = 1.2 Hz, *J_2_* = 8.8 Hz); 7.56 (*d*, 1H, *J* = 8.8 Hz); 7.94 (*d*, 1H, *J* = 8 Hz); 6.71 (*d,* 1H, *J* = 8 Hz); 3.91 (*s*, 3H); 3.87 (*s*, 3H); 4.16 (*s*, br, 2H). ^13^C-NMR (CDCl_3_) δ 105.32; 98.48; 117.58; 162.64; 138.81; 160.19; 113.75; 130.61; 120.47; 130.93; 113.91; 150.74; 55.55; 55.47; 189.70.

(*E*)-*1-(4-Aminophenyl)-3-(2,5-dimethoxyphenyl)prop-2-en-1-one* (**6**): orange crystals (1.58 g, 90%); m.p = 130–132 °C; Rf = 0.39 (*n*-hexane/ethyl acetate: 3/2); HRESI-MS [M+H]^+^ calculated for C_17_H_17_NO_3_ 283.1287, found 283.1284; IR (KBr, cm^−1^): 3445 and 3348 (-NH_2_), 1664 (C=O), 1607 (C=C_chain_), 1175 (C-O-C_aryl alkyl ether_); ^1^H-NMR (CDCl_3_) δ 8.05 (*d*, 1H, *J* = 15.6 Hz); 7.59 (*d*, 1H, *J* = 15.6 Hz); 7.19 (*d*, 1H, *J* = 8 Hz); 6.93 (*dd*, 1H, *J* = 8.8 Hz); 6.90 (*s*, 1H); 7.94 (*d*, 1H, *J* = 8.8 Hz); 6.71 (*d*, 1H, *J* = 8.8 Hz); 7,94 (*d*, 1H, *J* = 8.8 Hz); 3.89 (*s*, 3H); 3,84 (*s*, 3H); 4.19 (*s,* br, 2H). ^13^C-NMR (CDCl_3_) δ 138.45; 125.09; 123.22; 150.97; 116.65; 112.55; 153.23; 113.77; 131.09; 131.54; 113.93; 153.56; 56.19; 55.87; 188.70.

(*E*)-*1-(4-Aminophenyl)-3-phenylprop-2-en-1-one* (**7**): yellow crystals (0.59 g, 42%); m.p = 90–92 °C; Rf = 0.50 (*n*-hexane/ethyl acetate: 3/2); HRESI-MS [M+H]^+^ calculated for C_15_H_13_NO 224.1075, found 224.1073; IR (KBr, cm^−1^): 3339 and 3213 (-NH_2_), 1628 (C=O), 1603 (C=C_chain_); ^1^H-NMR (CDCl_3_) δ 7.81 (*d*, 1H, *J* = 15.6 Hz); 7.51 (*d*, 1H, *J* = 15.6 Hz); 7.65 (*m*, 1H); 7.43 (*m*, 1H); 7.96 (*d*, 1H, *J* = 8.8 Hz); 6.72 (*d*, 1H, *J* = 8.8 Hz); 4.21 (*s*, br, 2H). ^13^C-NMR (CDCl_3_) δ 143.1; 122.1; 135.4; 128.5; 128.9; 130.1; 128.3; 131.1; 113.9; 151.1; 188.16.

(*E*)*-3-(2-Methoxyphenyl)-1-phenylprop-2-en-1-one* (**8**): yellow oily liquid (0.52 g, 27%); Rf = 0.78 (CHCl_3_); ESI-MS [M+H]^+^ 239.2; IR (KBr, cm^−1^) 1661 (C=O), 1601 (C=C_chain_); ^1^H-NMR (CDCl_3_) δ 8.21 (*d*, 1H, *J* = 17.2 Hz); 7.65 (*d*, 1H, *J* = 17.2 Hz); 8.05 (*s*, 2H); 7.46 (*d*, 2H); 7.62 (*s*, 1H); 7.53 (*d*, 1H); 7.33 (*t*, 1H); 6.96 (*t*, 1H); 6.88 (*d*, 1H); 3.78 (*s*, 3H); ^13^C-NMR (CDCl_3_) δ 128.38; 128.45; 128.98; 138.31; 190.71; 122.42; 140.13; 126.61; 158.62; 111.10; 131.76; 120.60; 132.49; 55.32. 

(*E*)-*3-(3-Methoxyphenyl)-1-phenylprop-2-en-1-one* (**9**): yellow oily liquid (1.31 g, 86%); Rf = 0.47 (*n*-hexane/ethyl acetate = 3/2); ESI-MS [M+H]^+^ 293.3; IR (KBr, cm^−1^) 1663 (C=O), 1605 (C=C_chain_); ^1^H-NMR (CDCl_3_) δ 7.54 (*d*, 1H, *J* = 15.7 Hz); 7.88 (*d*, 1H, *J* = 15.7 Hz); 8.02 (*d*, 2H); 7.46 (*d*, 2H); 7.55 (*s*, 1H); 7.13 (*s*, 1H); 6.92 (*d*, 1H); 7.28 (*t*, 1H); 7.18 (*d*, 1H); 3.71 (*s*, 3H); ^13^C-NMR (CDCl_3_) δ 137.9; 128.4; 128.5; 132.7; 190.1; 122.0; 144.5; 136.0; 113.4; 159.8; 116.1; 129.8; 120.9; 55.1.

(*E*)-*3-(4-Methoxyphenyl)-1-phenylprop-2-en-1-one* (**10**): pale yellow crystals (1.09 g, 57%); m.p = 118–120 °C; Rf = 0.62 (CHCl_3_); ESI-MS [M+H]^+^ 239.3; IR (KBr, cm^−1^) 1657 (C=O), 1599 (C=C_chain_); ^1^H-NMR (CDCl_3_) δ 7.77 (*d*, 1H, *J* = 13.45 Hz); 7.42 (*d*, 1H, *J* = 13.45Hz); 7.92 (*d*, 2H); 7.47 (*d*, 2H); 7.54 (*s*, 1H); 7.56 (*d*, 2H, *J* = 6.72 Hz); 6.92 (*d*, 2H, *J* = 6.72 Hz); 3.83 (*s*, 3H); ^13^C-NMR (CDCl_3_) δ 138.6; 128.7; 130.4; 132.7; 190.7; 119.9; 144.9; 127.7; 128.6; 114.6; 161.8; 55.6.

(*E*)-*3-(2,3-Dimethoxyphenyl)-1-phenylprop-2-en-1-one* (**11**): yellow oily liquid (0.89 g, 41%); ESI-MS [M+H]^+^ 269.2; IR (KBr, cm^−1^) 1663 (C=O), 1603 (C=C_chain_); ^1^H-NMR (CDCl_3_) δ 8.11 (*d*, 1H, *J* = 15 Hz); 7.57 (*d*, 1H, *J* = 15 Hz); 8.06 (*d*, 2H); 7.43 (*t*, 2H); 7.53 (*d*, 1H); 6.89 (*d*, 1H); 7.03 (*t*, 1H); 7.23 (*d*, 1H); 3.77 (*s*, 3H); 3.81 (*s*, 3H); ^13^C-NMR (CDCl_3_) δ 138.1; 128.5; 128.4; 132.7; 190.5; 124.2; 139.4; 128.9; 148.8; 153.1; 114.14; 123.2; 119.4; 55.7; 61.2.

(*E*)-*3-(2,4-Dimethoxyphenyl)-1-phenylprop-2-en-1-one* (**12**): yellow crystals (1.10 g, 51%); m.p = 172–174 °C; Rf = 0.52 (*n*-hexane/CHCl_3_ = 3/7); ESI-MS [M+H]^+^ 269.3; IR (KBr, cm^−1^) 1651 (C=O), 1581 (C=C_chain_); ^1^H-NMR (CDCl_3_) δ 8.05 (*d*, 1H, *J* = 15.55 Hz); 5.22 (*d*, 1H, *J* = 15.55 Hz); 8.02 (*d*, 2H); 7.50 (*d*, 2H); 7.56 (*d*, 1H); 6.46 (*d*, 1H); 6.51 (*d*, 1H); 6.53 (*d*, 1H); 3.84 (*s*, 3H); 3.88 (*s*, 3H); ^13^C-NMR (CDCl_3_) δ 139.06; 128.68; 128.66; 132.51; 191.46; 120.63; 140.78; 117.33; 131.20; 105.58; 163.23; 98.65; 160.62; 55.72; 55.77.

(*E*)-*3-(2,5-Dimethoxyphenyl)-1-phenylprop-2-en-1-one* (**13**): yellow oily liquid (1.55 g, 72%); Rf = 0.39 (*n*-hexane/ethyl acetate = 3/2); ESI-MS [M+H]^+^ 269.2; IR (KBr, cm^−1^) 1660 (C=O), 1601 (C=C_chain_); ^1^H-NMR (CDCl_3_) δ 8.08 (*d*, 1H, *J* = 15.68 Hz); 7.53 (*d*, 1H, *J* = 15.68 Hz); 7.95 (*d*, 2H); 7.42 (*d*, 2H); 7.12 (*d*, 1H); 7.48 (*d*, 1H, *J* = 7.84 Hz); 6.75 (*d*, 1H, *J* = 7.84 Hz); 6.85 (*d*, 1H); 3.70 (*s*, 3H); 3.74 (*s*, 3H); ^13^C-NMR (CDCl_3_) δ 138.63; 128.74; 128.41; 132.54; 191.30; 122.66; 140.38; 124.65; 117.13; 153.67; 113.54; 112.29; 153.53; 56.30; 56.03.

(*E*)-*1-(4-Bromophenyl)-3-(4-methoxyphenyl)prop-2-en-1-one* (**14**): pale yellow crystals (0.64 g, 68%); m.p = 170–172 °C; Rf = 0.62 (*n*-hexane/ethyl acetate = 8/2); ESI-MS [M+H]^+^ 317.3 and 319.2; IR (KBr, cm^−1^) 1655 (C=O), 1593 (C=C_chain_); ^1^H-NMR (CDCl_3_) δ 7.77 (*d*, 1H, *J* = 18 Hz); 7.46 (*d*, 1H, *J* = 18 Hz); 7.85 (*d*, 2H, *J* = 9 Hz); 7.63 (*d*, 2H, *J* = 9 Hz); 7.61 (*d*, 2H, *J* = 9 Hz); 6.94 (*d*, 2H, *J* = 9 Hz); 3.84 (*s*, 3H); ^13^C-NMR (CDCl_3_) δ 127.8; 132.07; 130.60; 137.4; 189.6; 119.3; 145.5; 127.6; 130.2; 114.68; 162.06; 55.7.

(*E*)-*1-(4-Bromophenyl)-3-(2,4-dimethoxyphenyl)prop-2-en-1-one* (**15**): yellow crystals (0.91 g, 87%); m.p = 133–135; Rf = 0.50 (*n*-hexane/ethyl acetate = 9/1); ESI-MS [M+H]^+^ 347.3 and 349.3; IR (KBr, cm^−1^) 1651 (C=O), 1587 (C=C_chain_); ^1^H-NMR (CDCl_3_) δ 8.02 (*d*, 1H, *J* = 15.8 Hz); 7.46 (*d*, 1H, *J* = 15.8 Hz); 7.60 (*d*, 2H, *J* = 8.5 Hz); 7.84 (*d*, 2H, *J* = 8.5 Hz); 6.45 (*s*, 1H); 6.51 (*dd*, 1H, *J_1_* = 8.6 and *J_2_* = 2.2 Hz); 7.54 (*d*, 1H, *J* = 8.6 Hz); 3.83 (*s*, 3H); 3.88 (*s*, 3H); ^13^C-NMR (CDCl_3_) δ 137.8; 130.2; 131.9; 127.5; 190.3; 120.0; 141.4; 117.1; 131.4; 105.7; 163.8; 98.6; 160.7; 55.7; 55.8.

(*E*)-*1-(4-Bromophenyl)-3-(2,5-dimethoxyphenyl)prop-2-en-1-one* (**16**): pale yellow crystals (1.02 g, 98%); m.p = 112–114 °C, Rf = 0.68 (CHCl_3_/ethyl acetate = 6/4); ESI-MS [M+H]^+^ 347.1 and 349.2; IR (KBr, cm^−1^) 1666 (C=O), 1599 (C=C_chain_); ^1^H-NMR (CDCl_3_) δ 8.05 (*d*, 1H, *J* = 15.9 Hz); 7.56 (*d*, 1H, *J* = 15.9 Hz); 7.86 (*d*, 2H, *J* = 9.09 Hz); 6.49 (*d*, 1H, *J* = 9.09 Hz); 6.95 (*dd*, 1H, *J_1_* = 9.09 and *J_2_* = 3 Hz); 7.18 (*d*, 1H, *J* = 3 Hz); 3.61 (s, 3H); 3.66 (s, 3H); ^13^C-NMR (CDCl_3_) δ 137.37; 135.68; 132.06; 130.31; 190.26; 122.78; 141.02; 127.85; 153.60; 117.68; 114.06; 153.69; 112.65; 56.07; 56.32.

(*E*)-*1,3-diphenyl-2-propen-1-one* (**17**): pale yellow crystals (1.18 g, 90%); m.p = 57–59 °C; ESI-MS [M+H]^+^ 209.3; IR (KBr, cm^−1^) 1664 (C=O), 1607 and 1574 (C=C_chain_); ^1^H-NMR (CDCl_3_) δ 7.99–8.04 (*m*, 2H); 7.81 (*d*, 1H, *J* = 18.18 Hz); 7.55 (*d*, 1H, *J* = 18.18 Hz); 7,61–7.66 (*m*, 3H); 7.48–7.52 (*m*, 2H); 7.38–7.42 (*m*, 3H); ^13^C-NMR (CDCl_3_) δ 191.0; 145.08; 133.38; 135.6; 133.1; 129.1; 128.8; 128.7; 128.6; 122.2.

### 3.3. Preparation of of PfFNR and PfFd in E. coli

Production and purification procedure of *Pf*Fd and *Pf*FNR from recombinant *E. coli* was carried out according to the protocol reported by Kimata-Ariga *et al.* [7]. Enzymatic activity analysis of NADPH-dependent ferredoxin reduction by FNR was performed as reported by Onda *et al.* [25] with little modification. Shortly, enzymatic analysis was performed under the following reaction conditions: solution A (200 µM Cyt-c, 3 mM G6P, 3 U/mL G6PDH, 100 mM NaCl, 50 mM tris-HCl pH 7.5, 25 nM FNR), *Pf*Fd in concentration 0–5 µM *Pf*Fd, and 200 mM NADPH.

### 3.4. Inhibitory Activity Assay

Inhibition activity of the electron transfer from *Pf*FNR to *Pf*Fd was determined by using a chain reaction with addition of Cyt-c as an electron acceptor (Equation (1)). The inhibition test was performed as reported by Onda *et al.* [25]. The solution of prepared compound (4 µL, 100 µM), NADPH (4 µL, 200 µM), and *Pf*Fd (12 µL, 1 mM) were mixed in 96-well plate, then solution A (180 µL, composed of 200 µM Cyt-c, 3 mM G6P, 3 U/mL of G6PDH, 100 mM NaCl, 50 mM Tris-HCl pH 7.5, and 25 nM *Pf*FNR) was added to the reaction mixture and mixed well. The rate of absorbance change was then observed for 5 min in 10 second intervals at 550 nm using a microplate reader. 

### 3.5. Ligand Docking Analysis

The ligand docking pose was analyzed through molecular docking experiments. The 3D structure of *Pf*Fd used as docking target was downloaded from the Protein Data Bank (access code 1IUE) and the structure of maize Fd-FNR was downloaded from Protein Data Bank (access code 1GAQ). Preparation of ligand and macromolecule was performed using AutoDock Tools 1.5.6 [26], whereas the docking experiment was carried out using AutoDock4 [27]. The dimension of the gridbox was made so that it covered the binding site of *Pf*FNR involving to amino acid residues involved in the *Pf*FNR-*Pf*Fd interaction as reported by Kimata-Ariga *et al.* [7]. Docking results were visualized by the PyMol program.

## 4. Conclusions 

In conclusion, we have designed and synthesized in good to excellent yield chalcone derivatives using Claisen-Schmidt reactions. The methoxyamino chalcone derivatives displayed promising antimalarial activity. Based on the docking results, the synthesized chalcones preferred to interact with FNR by electrostatic interaction through salt bridges between the amino groups of the synthesized chalcones and the carboxyl groups of active amino acid residues of FNR rather than with Fd.
